# A Neurological Surgery Care Protocol for the LGBTQIA+ Community

**DOI:** 10.7759/cureus.52005

**Published:** 2024-01-10

**Authors:** José A Álvarez-Castro, Fernando Castro-Soto, Jonathan Ramos-Escalante, Miguel A Adame-Eslava, Andrea García-Bitar, Daniel Ballesteros-Herrera, Michel G Mondragon-Soto, Jorge Pastrana-Vivero, José O Santellán-Hernández, Sonia I Mejía-Pérez

**Affiliations:** 1 Department of Neurosurgical Oncology, National Institute of Neurology and Neurosurgery, Mexico City, MEX; 2 Department of Neurosurgery, National Institute of Neurology and Neurosurgery, Mexico City, MEX

**Keywords:** transgender medicine, best practice, lgbt+q, neurosurgery training, inclusion and diversity

## Abstract

This research aims to propose a neurological surgery care protocol for the lesbian, gay, bisexual, transgender, queer, questioning, intersex, or asexual (LGBTQIA+) community. In recent years, people belonging to the LGBTQIA+ community have started to come out and express their identity due to growing awareness and various factors like the implementation of legal protections and rights in several countries; it is well documented in the literature that this community faces unique health needs as well as barriers and inequalities in healthcare. The lack of tailored training for medical specialists affects the level of quality and access to medical care for these individuals, and neurosurgical care is no exception.

This literature review included studies in scientific journals and articles discussing problems, best practices, and gaps in the existing neurological surgical care protocols for LGBTQIA+ people. Accordingly, it highlights shared challenges such as healthcare-related difficulties, communication barriers, discrimination, and stigmatization. The primary aim is to create a safe and respectful care environment that ensures fair medical treatment to all patients regardless of their sexual orientation or gender identity. The review sheds light on the need for inclusive and sensitive neurosurgical care to improve clinical outcomes and the experience of patients belonging to the LGBTQIA+ community, thereby ensuring an environment of dignified treatment and satisfactory recovery from neurosurgical events.

## Introduction and background

Recent research has indicated that individuals who identify as lesbian, gay, bisexual, transgender, queer, questioning, intersex, or asexual (LGBTQIA+) have distinct health needs and require a specific healthcare approach [1]. The acronym encompasses various sexual or gender identity labels that individuals embrace and find meaningful [2]. Besides the identities covered by the letters in the abbreviation, the "+" signifies the inclusion of other evolving and diverse gender and sexual identities [3]. Individuals belonging to the LGBTQIA+ community experience both common and unique hurdles to obtaining optimum quality of healthcare. According to the World Health Organization (WHO), these obstacles are mainly associated with their sexual orientation, gender identity, and expression; they frequently experience limited access to healthcare, as well as stigma, and prejudice, and carry a disproportionate burden of negative physical and mental effects [4].

A lack of training in this area has had a significant impact on the quality of care and proper provision of healthcare services to these individuals. According to a survey conducted in Washington, DC, 68% of sexual minority youth did not disclose their sexual orientation to healthcare professionals, and 90% expressed worry about providing this information to their physicians [5]. Another research study emphasized the need to report sexual orientation to health professionals since these factors negatively influenced the experiences of LGBTQIA+ individuals regarding healthcare delivery [6]. Healthcare personnel, particularly highly trained experts such as neurosurgeons, must expressly acquire skills and knowledge in caring for LGBTQIA+ patients. Addressing systemic barriers and healthcare disparities is necessary to ensure equitable care [7].

Several terms have been introduced to establish a common language and clarify the experiences and realities of the LGBTQIA+ community. One key distinction is between sex (which refers to the biological, genetic, hormonal, anatomical, and physiological characteristics that determine whether a person is male or female at birth) and gender (which refers to the outward expressions of gender, such as postures, clothing choices, gestures, language patterns, behavior, and social interaction) [8]. Furthermore, sexual orientation must be defined as an individual's ability to feel or not feel attraction to others of the same or different gender, more than one gender or identity, including the ability to participate in personal connections with those individuals. Table 1 provides a summary of the LGBTQ+ vocabulary to enhance the understanding of these concepts [9].

**Table 1:** Summary of LGBTQ+ vocabulary

Term	Definition
Agender	An individual who does not identify with a specific gender [8]
Asexual	An individual who experiences minimal or no sexual attraction [8]
Bisexual	People having emotional and/or sexual attraction towards individuals of both the same gender and different genders [2]
Cisgender	People whose gender identity matches the sex they were assigned at birth [9]
Gay	An individual who is primarily or exclusively attracted to individuals of the same gender. This term is commonly used to describe men's sexuality [8]
Homosexual	Sexual, emotional, and affective attraction to individuals of the same gender. It is considered an outdated term, and "gay" is the preferred term [9]
Intersex	An individual whose biological sex does not fit within the typical definitions of male or female. Their sex characteristics may fall outside or between the male and female binary [8]
Lesbian	A woman who experiences primarily or exclusively romantic and/or sexual attraction to other women [8]
Non-binary	Individuals who do not identify strictly as male or female. This may be because they identify as both masculine and feminine, or neither, or their gender identity does not conform to societal norms regarding sex and gender [1]
Pansexual	An individual who experiences romantic and/or sexual attraction to others regardless of their sex, gender, gender identity, sexual orientation, or roles [9]
Queer	An umbrella term that includes individuals in the LGBTQ+ community who do not identify as heterosexual or cisgender. It is used to describe people who reject the gender assigned at birth and do not identify with any other gender [8]
Transgender	An individual whose current gender identity differs from their assigned sex at birth [9]
Transition	The process of adopting a gender role that differs from the one typically associated with the sex assigned at birth. This may involve social and/or medical changes [8]

This review aims to propose a protocol for neurosurgical care for individuals belonging to the LGBTQIA+ community, with the goal of ensuring equitable medical treatment to all regardless of their sexual orientation or gender identity; additionally, we provide specific recommendations on how to adapt medical care to meet the needs of LGBTQIA+ individuals at each stage of their medical treatment, including postoperative follow-up.

## Review

Objectives

The major goal of this review was to examine current academic publications and research studies linked to the healthcare requirements and experiences of people identifying as LGBTQIA+ who are treated for neurosurgical disorders. It focused on the medical, psychological, and sociocultural aspects that may impact the quality of care and treatment outcomes in the setting of neurological surgery. It also aimed to identify gaps in current knowledge and practices, as well as offer insight into areas where changes and personalized procedures are required to guarantee fair, inclusive, and sensitive healthcare for this unique group.

The secondary goal was to consolidate relevant information to provide recommendations to healthcare practitioners and researchers on the obstacles and potential in establishing customized care protocols for LGBTQIA+ patients undergoing neurological surgery. The study intended to contribute to the creation of evidence-based guidelines and recommendations that prioritize patient well-being and satisfaction through the analysis of relevant literature.

Methodology

This literature review was conducted to explore and summarize current information on neurological surgery care for the LGBTQIA+ community. The search strategy focused on open-access publications, peer-reviewed journals, and pertinent gray literature. The search technique included scouring reliable academic sources such as PubMed, Scopus, and Google Scholar for research and scholarly works that addressed the convergence of neurological surgery and healthcare inequities in the LGBTQIA+ population. To guarantee the search's inclusiveness, keywords such as "neurological surgery," "LGBTQIA+," "healthcare protocol," and similar phrases were included in various combinations.

Articles included were published within the previous decade, written in English, and presented insights into the problems, best practices, and gaps in the existing neurological surgical care protocols for LGBTQIA+ people. This method enabled a rigorous and transparent procedure, and it allowed for a full examination of relevant literature contributing to the synthesis of evidence-based recommendations for improving care practices in this unique healthcare environment.

Epidemiology 

A global study was undertaken in 2023 to investigate the epidemiology of the LGBTQIA+ population. The survey included 22,514 people aged 16-74 years from 30 different countries. The results demonstrated that 3% of respondents identified as lesbian or homosexual, 4% as bisexual, 1% as pansexual, and 1% as asexual. Males were more likely to identify as homosexual (4%) than females were to identify as lesbian (1%). On a global scale, Spain had the largest proportion of homosexual or lesbian respondents (6%), while Brazil and the Netherlands had the highest proportion of bisexual respondents (7%) [10].

**Figure 1:**
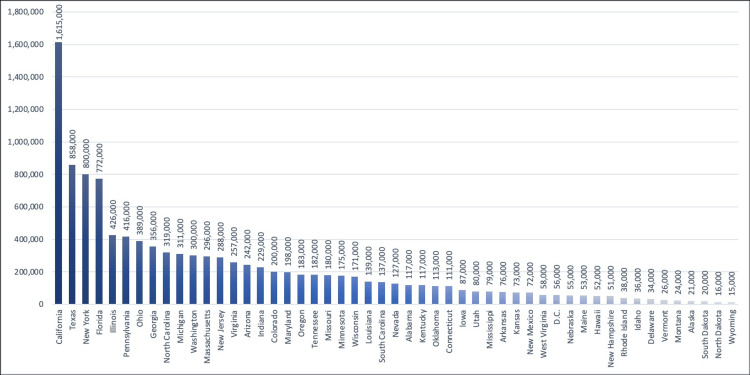
Adult LGBTQIA+ population in the US by state Data extracted from Williams Institute’s LGBT Data & Demographics Interactive, 2020 [11]

The proportion of individuals aged 18 years and above who identify as LGBTQIA+ has increased in the past 10 years. In a survey conducted in 2017, 4.5% of the adult population in the United States was identified as LGBTQIA+; the states with the largest proportions of LGBTQIA+ people were California, New York, and Texas, whereas Wyoming and North Dakota had the lowest numbers of LGBTQIA+ individuals (Figure 1) [11].

**Figure 2:**
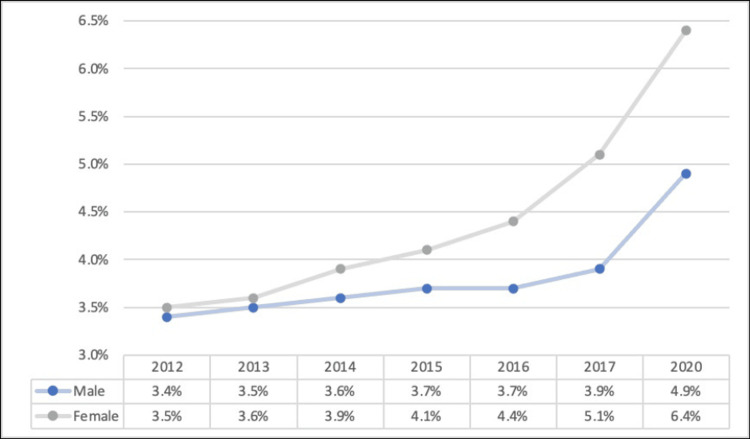
Percentage of people in the US identifying as LGBTQIA+ by gender Data extracted from Gallup telephone polls, 2022 [15]

However, another study by the Statista Research Department showed that 7.1% of people in the U.S. identified themselves as part of the LGBTQIA+ community. Meanwhile, individuals born between 1997 and 2004 tended to identify as part of the LGBTQIA+ at a greater rate, with nearly one in every five young people falling into this group, and females were more likely than males to identify as members of the LGBTQIA+ group (Figure 2) [12-15].

**Figure 3:**
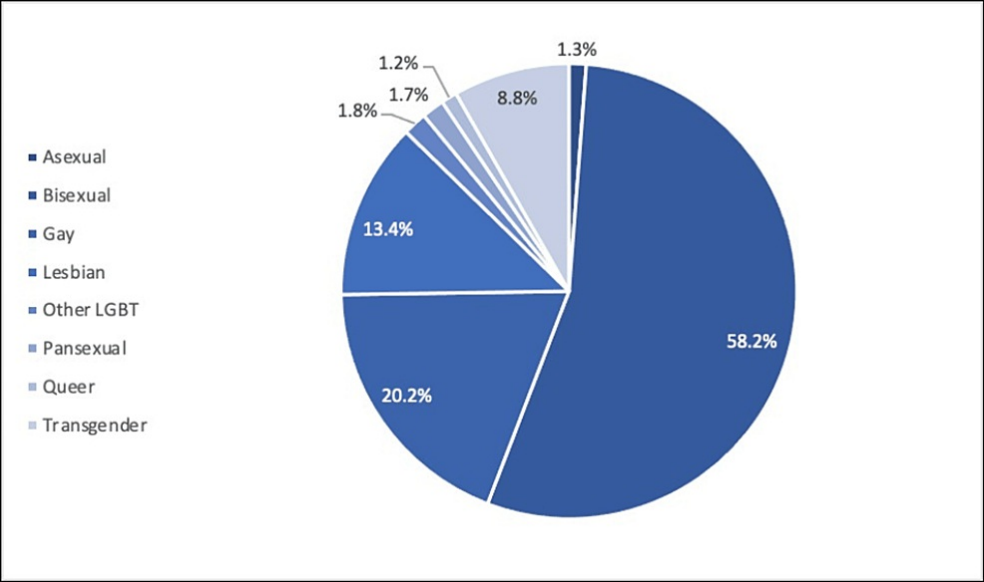
Gender identity or sexual orientation types among the US LGBTQIA+ adults Data extracted from Gallup telephone polls, 2022 [15]

The proportion of the community remained stable at 7.2% of the general population, with most people identifying as bisexuals (4.2%), gay (1.4%), lesbian (1%), and transgender (0.6%). These estimations are contingent on the LGBTQIA+ community's willingness to reveal their sexual orientation and gender identity. The data may be biased since the population is not yet fully prepared to freely reveal their identity (Figure 3) [12-15].

Cultural competency, economic literacy, and sensitivity

Personal, social, economic, and environmental factors contribute to individual and overall well-being as social determinants of health [16]. LGBTQIA+ individuals experience similar health injustices and challenges as other disadvantaged groups. These include aspects related to income, educational achievement, and racial and ethnic backgrounds [17]. Stigmas and biases embedded in society can impair healthcare experiences, while cultural obstacles may impact their ability to seek timely care, interact honestly with physicians, and obtain appropriate treatment. Lack of awareness and sensitivity toward the needs and experiences of LGBTQIA+ patients can have a substantial influence on the quality of care offered in the field of neurosurgery, where decision-making is crucial, and the physician-patient relationship is paramount. 

Economic determinants can have a significant impact on the acquisition of healthcare insurance among the LGBTQIA+ population. For instance, differences in economic resources resulting from job selection and discrimination can influence the earning potential of sexual minorities [18]. Furthermore, research has shown that people who identify as bisexual or transgender, are over the age of 80 years, or have HIV are more likely to face economic insecurity, which affects their ability to access healthcare services and contributes to health disparities [19].

Moreover, LGBTQIA+ individuals experience significant hurdles in terms of acquiring health insurance coverage. Historically, they were much less likely than non-LGBTQIA+ people to have health coverage in 2013 and were more likely to have issues related to receiving critical medical care. when compared to 2019 data, coverage rates for LGBTQIA+ adults were equivalent to those of non-LGBTQIA+ adults, although considerable gaps in access remained [20].

Because of the prohibitive costs associated with neurosurgical care, these economic determinants represent a significant barrier for many LGBTQIA+ people who face economic disparities and financial difficulties, resulting in inaccessibility to necessary procedures and treatments, negatively affecting their health and well-being.

Strategies to set up an inclusive and welcoming environment for LGBTQIA+ patients

Safe clinical environments are critical in providing care for the LGTQIA+ community; while many patients have pleasant contact with physicians, many individuals postpone or avoid medical care due to stigma, social isolation, and prejudice [21]. Several studies have shown that patients faced prejudice and expressed concern about their doctor's reaction to their gender identity, leading them to actively seek providers who are at ease with the LGBTQIA+ community [22].

Within the LGBTQIA+ healthcare delivery model, several actions have been implemented to reduce stigma and promote empowerment and equal care. These include:

Paying more attention to physical and virtual environments through inclusive language (both in-person and on forms) and featuring diverse LGBTQIA+ patients in advertisements and artwork.

Hiring providers who offer services to meet the preventive healthcare and health promotion needs of the LGBTQIA+ community.

Indicating in the provider's details their desire to be recognized as LGBTQIA+-competent/friendly and enrolling in the Health Professionals Advancing LGBTQIA+ Equality provider directory.

Providers and staff can choose to incorporate identifiers into their attire or office space, such as a selection of books, stickers, and posters, to demonstrate a welcoming environment for LGBTQIA+ patients.

Establishing healthcare facilities within or near areas with a concentration of LGBTQIA+ households, businesses, and community centers.

By ensuring competent and LGBTQIA+-friendly environments and providers, clinics can enhance their care for the LGBTQIA+ community and create welcoming spaces that are free from discrimination and judgment [21].

Developing admission and assessment procedures

In 2011, the Institute of Medicine and the Joint Commission recommended that data on patients' sexual orientation and gender identity be collected and documented in healthcare settings as an essential step in providing patient-centered care [23]. Routine data collection helps healthcare institutions track, monitor, and manage inequities in their LGBTQIA+ patient care and enhance the quality of their treatment [24]. 

Clinicians should ask questions about sexual orientation, behavior, and gender identity when addressing a patient for the first time. They can begin with an open inquiry like “Tell me a little bit about yourself” and use neutral wording like “Do you have a partner?” instead of asking pointed questions like “Are you married?”, which normally relates to heterosexual partnerships for most people.

Assuring the patient and earning his or her trust is paramount, and it should be explained that these questions are asked of all patients and such information is required for ensuring the best possible treatment. Many patients are open to sharing such information, particularly once they realize why these queries are posed. Once an open connection is established with the patients and they are assured that they are in a safe and comfortable space, neurosurgeons should provide treatment that deals with the unique health challenges and inequities that LGBTQIA+ people encounter [25].

Ensuring all staff are trained to use inclusive language

All staff members who interact with patients should learn how to communicate effectively and respectfully with LGBTQIA+ patients. They will also require training to provide adequate care for LGBTQIA+ patients, including the consistent use of correct patient data such as names and pronouns. Staff training programs can be incorporated into new employee orientation and repeated annually [24].

Healthcare personnel should ask open-ended questions and avoid assuming the person seeking care is heterosexual. In addition, the use of gender-neutral pronouns is recommended; they should inquire about how the patient identifies in terms of gender or sexual orientation. Likewise, physicians should be aware of the various aspects of the health of sexual minorities and understand both the unique health needs of the LGBTQIA+ community and the stigma, discrimination, and violence faced by many individuals in this community [21]. It is recommended to avoid using nicknames, gestures of rejection, or any degrading expressions that discriminate or make the patient feel uncomfortable.

Hostile questioning related to gender identity or sexual orientation should always be avoided, and equality in the treatment of same-sex and lesbian maternal families should be ensured; the approach should be objective, respectful, and empathetic, and patients and their families should be treated with dignity. The confidentiality of data related to a person's health condition or medical diagnosis, as well as their sexual orientation, should be protected, ensuring that their information is not disclosed without authorization.

Neurosurgical planning and care

Due to the increasing number of individuals identifying as part of the LGBTQIA+ community, healthcare providers must be aware of specific considerations relevant to the neurosurgical perioperative process [7]. As a first step, physicians must heed the patient's gender identification by asking the patient about their preferred gender identity, and the sex assigned at birth. Inquiring about the patient's preferred name, pronouns, and terminology creates a conducive atmosphere to build trust and establish a channel of communication so that medically important information can be effortlessly provided [26-28].

In the LGBTQIA+ community, the incidence of certain sexually transmitted diseases is higher than in other groups, especially men who have sex with men and transgender women may be at an increased risk of HIV, human papillomavirus, hepatitis B, and methicillin-resistant *Staphylococcus aureus* infections [7,29].

Approximately 80% of transgender patients are currently receiving or are interested in receiving gender-affirming hormone therapy, which must be evaluated because some of these regimens are associated with surgical risks [30]. Transgender males may use testosterone undecanoate and parenteral options like testosterone enanthate and testosterone cypionate, as well as testosterone implants and transdermal gels. Transgender women receiving feminizing hormone therapy may be given ethinylestradiol and estradiol, estradiol valerate, transdermal estradiol, and antiandrogens like progesterone, medroxyprogesterone acetate, GnRH agonists, histrelin implants, spironolactone, and finasteride [31]. These regimens should not be stopped as these could put patients at risk of psychological and physical repercussions [32]. Moreover, treatment with hormones can result in certain changes in the blood chemistry of transgender men; hormone therapy may lead to increased levels of hemoglobin, aspartate transaminase, alanine aminotransferase, creatinine, total cholesterol, triglycerides, and could cause iron deficiency and erythrocytosis [28,33,34].

Meanwhile, transgender women experience alterations in lab values, such as higher levels of lipids, triglycerides, total cholesterol, high-density lipoproteins, hemoglobin, red cells, and prolactin; and lower levels of hematocrit, alanine aminotransferase, aspartate transaminase, alkaline phosphatase, creatinine, low-density lipoproteins, and calcium [28,34,35]. It is crucial to consider the possibility of prolactinoma if symptoms such as headache, visual alterations, or galactorrhea are present, and imaging tests are recommended [36].

In transgender women undergoing gender-affirming hormone therapy, there is an elevated risk of venous thromboembolism (VTE), especially when accompanied by other risk factors like smoking, alcohol consumption, sedentary lifestyle, overweight or obesity, and hypercoagulation disorders. Transgender women undergoing hormone therapy with ethinylestradiol are at a higher risk of having VTE [32]. The decision to administer anticoagulant prophylaxis should be based on each patient's Caprini score, which may aid in the clinician’s decision-making [37].

Neurosurgeons need to be aware of structural changes in the brain that are relevant and well-documented in the LGBTQIA+ population. In transgender men, there is an observed increase in the size of the parietal and temporal cortex, as well as higher values of fractional anisotropy in the superficial longitudinal fasciculus, corticospinal tract, and forceps minor; gray matter volume in the cerebellum could be decreased, and there could be a decrease in lobar interhemispheric connections. Transgender women may experience an increase in the thickness of certain regions of the brain, including the insular, orbitofrontal, and medial occipital cortex. Both transgender men and women may have anatomical variances in the corpus callosum and sagittal medial plane, along with variations in the volume of the putamen; these differences are related more to their gender identity than the sex assigned at birth. Furthermore, changes in the volume and thickness of the cortex have also been observed [38]. 

Transgender patients who use hormone therapies may be at an increased risk of developing certain tumors in the nervous system. Transgender men have been reported to experience prolactinoma and somatomammotropinoma more often. In contrast, transgender women are at a higher risk of developing pituitary adenoma, meningioma, vestibular, schwannoma, and glioblastoma. Certain tumor concerns must be evaluated in perioperative settings for neurosurgical operations [38,39].

In a neurosurgical setting, the clotting function is a relevant aspect to consider when treating a transgender patient undergoing cross-sex hormone therapies. In transgender men, elevated levels of coagulation factors IX, hematocrit, and S protein may be observed when they are undergoing treatment with masculinizing hormones [40]. Conversely, transgender women undergoing cross-sex hormone treatment may exhibit higher levels of coagulation factors IX, XI, and fibrinogen, along with a lower hematocrit level. These factors should be considered when assessing the coagulation status of transgender patients in the preoperative stage (Table 2) [40].

**Table 2:** Preoperative considerations in transgender patients IM: intramuscular injection; po: oral route

Points to evaluate	Transgender men	Transgender women	Both
Medications used in hormone therapy	Dissolved testosterone crystals (gel and patch); testosterone cypionate (IM); testosterone enanthate (IM); testosterone undecanoate; progestin; medroxyprogesterone; linestreno [28]	Ethinyl estradiol (po); 17-ß-estradiol (po); medroxyprogesterone (po); transdermal patch with norethindrone; cyproterone; spironolactone; flutamide; triptorelin; progesterone [28]	
Risk factors	HIV, smoking, alcoholism, drug addiction, implants, hypercoagulability disorders, hepatitis B, pontine myelinolysis, myelopathy, peripheral neuropathy, and motor neuron syndromes due to HIV complications [29]		
Preoperative evaluation	Venous thrombosis ↑ D-dimer levels; risk assessment of bleeding and thrombosis using HEMORRHAGES, HAS-BLED, and Caprini scores [28]		
Changes in blood chemistry	↑ Hematocrit ↑ Hemoglobin ↑ Erythrocytes ↑ Creatinine ↑ Total cholesterol ↑ Triglycerides ↑ Transaminases ↑ Alkaline phosphatase [33]	↑ Triglycerides ↓ Calcium ↑ Total cholesterol ↓ Albumin ↑ HDL ↑ Erythrocytes ↓ Hematocrit ↓ Alkaline phosphatase ↓ Hemoglobin ↓ Creatinine ↓ Transaminases ↑ Concentration of hemoglobin ↑ Coagulation factors (II, VII, VIII, X, and fibrinogen) ↓ Antithrombin ↓ Protein S Hyperprolactinemia [33]	
Special considerations	Pregnancy test; erythrocytosis; hemochromatosis [28]	Risk of venous thrombosis with ethinylestradiol (po); anticoagulation [28]	Using patient identification format accompanied by physical examination; not stopping hormone therapy (emotional and physiological adverse effects); thromboprophylaxis (low molecular weight heparins) [28]
Structural changes in the nervous system	↑ Size of the parietal and temporal cortex ↑ Values of fractional anisotropy in the superficial longitudinal fasciculus, corticospinal tract, and minor forceps ↓ Gray matter volume in the cerebellum [38]	↑ Trigging in width of the insular cortex, orbitofrontal, and occipital medial. ↑ The volume of gray matter in the cerebellum [38]	↓ Nucleus accumbens size, left thalamus, right hippocampus, and right caudate nucleus; changes in crust width and volume [38]
Associated nervous system tumors	Prolactinoma; somatomammotropinoma [38]	Meningioma; non-functioning pituitary adenoma; prolactinoma; vestibular schwannoma; glioblastoma [38]	
Recommendations	Evaluate the presence of acromegaly [33]	Prophylactic anticoagulation; add one point to the Caprini scale and act according to the guidelines; hyperprolactinemia screening; order imaging studies if prolactin levels are >80 mcg or if there are symptoms such as headache, galactorrhea, and visual changes [40]	Comprehensive management with services such as psychiatry, social work, endocrinology, anesthesiology, and psychology [28]
Clot times	↑ Factor IX and hematocrit ↓ Factor II, XI ↑ Protein S [40]	↑ Factors IX, XI, and fibrinogen ↓ Protein C ↓ Hematocrit ↑ Free Protein S [40]	↑ Ratio of activated protein C; consider age, time of administration, and associated risk factors [40]

During surgery, transgender men who have not undergone gender-affirming surgery may use breast binders or other garments that could potentially restrict respiration, causing severe respiratory complications. Transgender women may have undergone laryngoplasty or chondroplasty (surgical procedures involving the larynx or cartilage), and this could prove to be a concern when installing orotracheal intubation [28]. In the operating room, it is important to prioritize patient privacy by minimizing the presence of unnecessary personnel in the area to create a respectful and confidential environment for transgender patients [28]. In cases where transgender patients who have undergone gender-affirming surgery require a vesical catheter, it is recommended to consult with urology or another specialized service that is well-versed in the urogenital anatomy in relation to that surgery. 

When caring for transgender patients who are HIV-positive, standard precautions to prevent the transmission of HIV should be taken and the safety of the patient and healthcare providers involved should be ensured. Being aware of the medications taken by patients is critical to avoid potential drug interactions. HIV-positive patients who receive antiviral treatment may experience drug interactions with certain hypnotics, anxiolytics, and antibiotics [28]. Individuals taking feminizing hormone therapy may encounter interactions when taking antiseizure medications, corticosteroids, and neuromuscular blockers, such as succinylcholine; transoperative neuromuscular monitoring should be considered when neurosurgical procedures involve neuromuscular blockers (Table 3) [35].

**Table 3:** Intraoperative considerations in transgender patients

Points to evaluate	Transgender men	Transgender women			Both
Anesthesia	Molding plants that cause respiratory restriction [28]	Consider airway attachments during intubation in patients with pharyngoplasty and achondroplasty [28]			Caution in doses of propofol and remifentanil. Consider smoking and drug addiction. Intraoperative anesthesia administration according to standard guidelines [28]
Special considerations	Minimize unnecessary staff inside the operating room. Consider the size of the urinary catheter or consult urology with experience in the anatomy of transgender patients. Maintain standard precautions in case of HIV (+) patients [28]				
Drug interactions in anesthesia	Antivirals in HIV, sedatives, hypnotics, anxiolytics, and antibiotics [28]		Antivirals in HIV, sedatives, hypnotics, anxiolytics, and antibiotics. Anticonvulsants, corticosteroids, neuromuscular blockers (succinylcholine) [28]	Administration of medicines per standard practice [28]	
Recommendations	Consider neuromuscular monitoring in the use of neuromuscular blockers [33]			In cases of venous thrombosis, anticoagulants should be administered orally [33]	

In the postoperative setting, various parameters should be considered, including wound healing, pain management, and potential complications. In transgender patients, the process of scar formation is heavily influenced by the patient's risk factors. Keloid and hypertrophic scars can have significant psychosocial implications, and hence these require appropriate wound care. In addition, some patients may experience postoperative chronic pain, which can be aggravated by drug-induced osteoporosis [41]. Healthcare professionals must recognize that this population is at a higher risk and exhibits a greater incidence and prevalence of mental health disorders, including anxiety, depression, and suicide rates; surgical treatment increases the symptoms experienced by these individuals and may lead to extended hospital stays [28].

Throughout the preoperative and postoperative stages, the patient's privacy must be respected, and proper bed and room must be provided depending on the patient's gender identification. If possible, providing private rooms should be considered [33]; other measures, such as administering antiemetic prophylaxis and implementing postoperative prophylaxis for deep venous thrombosis, must also be considered. It should be noted that the use of spironolactone may lead to orthostatic hypotension (Table 4) [42].

**Table 4:** Postoperative considerations in transgender patients CVD: cardiovascular disease

Points to evaluate	Transgender men		Transgender women		Both
Cicatrization	Proper wound care should be performed, as keloid or hypertrophic scarring has psychosocial repercussions [41]				
Pain	Chronic postoperative pain from drug-induced osteoporosis [33]				
Complications	Exacerbation of mental illnesses and extended hospitalization [33]				
Recommendations	Risk of CVD due to polycythemia vera; promote vitamin D and calcium intake [33]	Antiemetic prophylaxis for deep vein thrombosis; orthostatic hypotension with the use of spironolactone [33]		Maintain patient privacy; assign bed and room based on patient's gender identity [33]	

Establishing a system to provide ongoing support to LGBTQIA+ patients after surgery

When caring for transgender neurosurgical patients, it is essential that the healthcare staff attending to them during their recovery consistently acknowledge their preferences regarding their name and pronouns; this is crucial for avoiding psychosocial stressors that can delay their recovery and reintegration into their regular environment. A better patient-physician connection has been linked to better adherence to medical advice following hospitalization [43]. The employment of gender-affirming hormone therapy has a neuroprotective effect in spinal cord injuries, especially estrogens that can reduce neuropathic pain. Nonetheless, the correct dose and route of administration must be ensured to obtain optimal benefits [44]. In the case of the gonadotropin-releasing hormone agonist regimen, the endocrinologist’s participation is essential to monitor bone mineral density and provide recommendations to reduce the risk of vertebral fractures following spinal surgery through calcium and vitamin D supplementation [45].

Transgender patients show a high incidence of anxiety and depression disorders aggravated by stressors such as surgical events and long-term hospitalization [28]. Moreover, the continuous administration of spironolactone to decrease testosterone effects in transgender females can produce a depressive mood [45]. Hence, a multidisciplinary approach is necessary, involving social workers, psychologists, and spiritual caregivers, to address all the factors involved in postoperative discomfort and to improve the recovery process and reintegration into their environment.

Facilitating connections between patients and LGBTQIA+ resources and support groups

Patients with LGBTQIA+ identities and neurological conditions often change treating physicians, resulting in loss of follow-up, and they are rarely referred to mental health services when compared to cisgender individuals. Moreover, they belong to an economically active population highly vulnerable to financial disparities and may experience higher rates of unemployment and poverty, particularly among those of African American descent.

Socially, they often face challenges in their family relationships due to the familial rejection of their identity; therefore, during the recovery period, these patients need an inclusive support network. It has been reported that the delay in seeking rehabilitation services is often attributed to fear of discrimination based on previous experiences, affecting up to 70% of cases [43,44].

Continuous assessment and evaluation of the effectiveness of the protocol to meet the needs of LGBTQIA+ patients

Recent studies have found that 43% of neurology specialists do not consider recognizing sexual orientation and gender identity as social determinant factors in the management of neurological conditions due to the challenges involved in patient care. Even today, 6% of residents in training disagree about providing equal treatment to other patients [43]. These limitations may be associated with insufficient medical training in gender diversity due to a lack of interest and expertise in the field [46]. As part of the social and cultural responsibility on the part of medical schools and healthcare institutions, it is recommended to incorporate content on gender diversity training in the formal curriculum to enhance a greater understanding and recognition of these patients.

One of the most studied neurosurgical entities within this patient population is the predisposition to develop meningiomas secondary to chronic use of progestogens as part of hormonal replacement therapy. Recent analyses report the benefits of implementing screening programs using MRI in these patients, given the likelihood of detecting small, multiple, asymptomatic lesions that may decrease in size after the discontinuation of progestin administration in up to 79% of cases [47]. This is one of the key preventive strategies that could facilitate the timely detection of lesions still amenable to conservative treatments in this population.

## Conclusions

This is the first literature review on neurosurgical care and treatment among the LGBTQIA+ community. Despite the availability of extensive studies on general healthcare, the evidence corresponding to transgender-specific needs is mostly limited to studies of short methodological scope and extrapolations of results from cisgender cases. Neurosurgical centers should proactively engage in gender education, foster multidisciplinary collaboration, and promote awareness of the unique needs of this community to enhance the quality of medical care and improve the quality of life for these patients. It is essential to recognize the value of drawing on the knowledge and experience of community organizers, especially within the current context in which health educators and public health professionals work. Overcoming obstacles to achieving general and healthcare equity requires a contextualized approach to addressing health and social issues, and community organizing can offer valuable strategies and lessons for public health professionals. By collaborating effectively with communities and using community power, we can work towards the shared goal of improving equity, both within our organizations and in society at large.
